# Incidence and admission rates for severe malaria and their impact on mortality in Africa

**DOI:** 10.1186/s12936-016-1650-6

**Published:** 2017-01-03

**Authors:** Flavia Camponovo, Caitlin A. Bever, Katya Galactionova, Thomas Smith, Melissa A. Penny

**Affiliations:** 1 Swiss Tropical and Public Health Institute, Basel, Switzerland; 2 University of Basel, Basel, Switzerland; 3 Institute for Disease Modeling, Bellevue, WA 98005 USA

## Abstract

**Background:**

Appropriate treatment of life-threatening *Plasmodium falciparum* malaria requires in-patient care. Although the proportion of severe cases accessing in-patient care in endemic settings strongly affects overall case fatality rates and thus disease burden, this proportion is generally unknown. At present, estimates of malaria mortality are driven by prevalence or overall clinical incidence data, ignoring differences in case fatality resulting from variations in access. Consequently, the overall impact of preventive interventions on disease burden have not been validly compared with those of improvements in access to case management or its quality.

**Methods:**

Using a simulation-based approach, severe malaria admission rates and the subsequent severe malaria disease and mortality rates for 41 malaria endemic countries of sub-Saharan Africa were estimated. Country differences in transmission and health care settings were captured by use of high spatial resolution data on demographics and falciparum malaria prevalence, as well as national level estimates of effective coverage of treatment for uncomplicated malaria. Reported and modelled estimates of cases, admissions and malaria deaths from the *World Malaria Report*, along with predicted burden from simulations, were combined to provide revised estimates of access to in-patient care and case fatality rates.

**Results:**

There is substantial variation between countries’ in-patient admission rates and estimated levels of case fatality rates. It was found that for many African countries, most patients admitted for in-patient treatment would not meet strict criteria for severe disease and that for some countries only a small proportion of the total severe cases are admitted. Estimates are highly sensitive to the assumed community case fatality rates. Re-estimation of national level malaria mortality rates suggests that there is substantial burden attributable to inefficient in-patient access and treatment of severe disease.

**Conclusions:**

The model-based methods proposed here offer a standardized approach to estimate the numbers of severe malaria cases and deaths based on national level reporting, allowing for coverage of both curative and preventive interventions. This makes possible direct comparisons of the potential benefits of scaling-up either category of interventions. The profound uncertainties around these estimates highlight the need for better data.

**Electronic supplementary material:**

The online version of this article (doi:10.1186/s12936-016-1650-6) contains supplementary material, which is available to authorized users.

## Background

Each year the prompt and effective treatment of *Plasmodium falciparum* malaria saves the lives of children across malaria endemic countries. Recent analysis has estimated that scale-up of vector control (insecticide-treated nets and indoor residual spraying) and artemisinin combination therapy have reduced malaria prevalence by 50% and clinical incidence by 40% in endemic Africa over the years 2000–2015 [1]. However, it is unclear how many deaths are prevented each year by the treatment of both uncomplicated and severe clinical malaria. Hospital case fatality rates for well-defined severe malaria are relatively well established [2, 3]. However these do not translate directly into estimates of the impact of effective management of severe disease on malaria mortality rates, for which only estimates based on expert opinion are available [4] and, to date, there are no good estimates of how these translate into numbers of malaria deaths averted.

The World Health Organization’s annual *World Malaria Report* (WMR) [5] provides information on country-specific numbers of cases and admissions reported by National Health Ministries. The admission rates vary enormously, raising the question of whether they can be interpreted in the same way for all countries. These statistics make no distinction between different levels of disease severity, and it would appear that in many countries large numbers of uncomplicated malaria patients are admitted as in-patients to health facilities.

The inclusion of uncomplicated malaria patients in statistics on malaria admission means that these numbers cannot be used uncritically to estimate the proportion of severely ill people that access such care. In general, population-based estimates must be used to estimate access rates, and recent Demographic and Health Surveys (DHS) and Malaria Indicator Surveys (MIS), have made much more data available on access to care for malaria [6–8]. These data demonstrate enormous variations between countries in access to treatment for uncomplicated malaria [9], but such surveys do not provide good estimates of severe malaria incidence in the community because it is a relatively infrequent acute disease, unlikely to be encountered at the exact time of a household visit, and cannot be reliably diagnosed from reported signs and symptoms. There are consequently no good direct estimates of the numbers of severe episodes in endemic countries that fail to access appropriate care. Several studies have found no better source of information on this than the 1996 review of McCombie [10], which is methodologically limited and now very outdated. Goodman et al. [11] summarized those results in 2000, proposing that on average 48% (with high and low estimates of 19 and 88%) of severe malaria cases in the sub-Saharan region are admitted, and several models have continued to use this value (or similar constant values [12, 13]) in the absence of any more reliable source [14, 15].

There are thus gaps in routine statistics on overall incidence of severe disease, on the corresponding care-gap, and its public health consequences. To contribute to filling these gaps this study proposes model-based methods to estimate the number of severe malaria cases occurring in each malaria endemic country in sub-Saharan Africa, the proportion admitted to in-patient care, and the corresponding public health burden. These methods rely on available national or geographic reported clinical and treatment data, as well as available risk and exposure information for each country. Specifically, the estimates are based on the following data or model inputs: (1) estimates of the distributions of transmission intensities based on the prevalence data assembled by the Malaria Atlas Project (MAP) [16]; (2) the effective coverage of treatment for uncomplicated malaria, estimated from survey data [9]; (3) national level reports of numbers of in-patient deaths and estimates of total deaths from WMR [5]; (4) models for severe disease incidence as allowing for the effects of treating uncomplicated disease, and calibrated by triangulating the relationships between severe disease, mortality, and transmission intensity [14]. By applying published estimates of case fatality rates among both in-patients and in the community we estimate the numbers of clinical episodes and deaths averted by case management of both uncomplicated and severe malaria and use these estimates to project the current burden of malaria in Africa. These estimates additionally indicate the public health impact that could be achieved by improving access to appropriate care for severe disease.

## Methods

### Data sources and notation

For each of the 41 countries in sub-Saharan Africa for which sufficient data was available, estimates for 2014 of the average incidence rate of both uncomplicated (*U*) and severe (*S*) clinical malaria, and the malaria specific (direct) mortality rate (*D*), were collated. In each case the estimates were disaggregated according to whether the event was as an in-patient (subscript *h*) or in the community (subscript *c*) or both combined (subscript *t*). The estimates were obtained from two sources, either as reported in WMR, denoted by accent $\widehat{}$, or calculated from simulation models of malaria epidemiology, denoted by accent $\bar{}$. Notation and variables are described in Table 1.

**Table 1:** Variables and parameter descriptions

Name	Description
*Variables*	
*U*	Incidence rate of uncomplicated clinical malaria (per 100,000 person per year)
*S*	Incidence rate of severe clinical malaria (per 100,000 person per year)
*C*	Incidence rate of total clinical malaria (C = U + S) (per 100,000 person per year)
*D*	Incidence rate of malaria mortality^a^ (per 100,000 person per year)
*μ*	Proportion of severe cases treated as in-patients (−)
*r*	Ratio of severe to total clinical cases for in-patients (−)
*Q* *R*	Case fatality rate (−) Estimated public health impact (as malaria mortality) averted with maximal improvement to admittance of severe disease patient (μ = 1) (per 100,000 person per year)
$\rho$	The overall ratio of the number of deaths per year in WMR ($\hat{D}_{t}$) [allowing for the national population (*N*)], to that predicted by *OpenMalaria* ($\bar{D}_{{t,\mu ,\bar{\varphi }_{1} }}$)
*Subscripts*	
*h*	Indicates in-patient event
*c*	Indicates event in community
*t*	Indicates total events
*PB*	Indicates prediction-biased estimate
*DA*	Indicates deaths-adjusted estimate
$\mu_{0}$	Indicates estimate used in *OpenMalaria* analysis for the proportion of severe cases treated as in-patients (usually $\mu_{0}$ = 0.48)
$\overline{\varphi }_{x}$	Indicates estimate calculated with the odds ratio of value $\overline{\varphi }_{x}$
*Accents*	
$\bar{}$	Indicates estimation from *OpenMalaria* simulations
$\hat{}$	Indicates estimation from WMR

^a^The mortality rates from WMR are compared to the direct mortality from *OpenMalaria*, the additional indirect mortality due to co-morbidities is not considered

WMR of 2015 [5] provides values for the 2014 overall incidence rate of clinical malaria in each country, $\hat{C}_{t}$, of the number of cases that are admitted, $\hat{C}_{h}$, of the overall mortality rates $\hat{D}_{t}$, and the rate of in-patients malaria death, $\hat{D}_{h}$.

The WMR is an important source of information on national trends in malaria incidence and mortality, but is reliant on hospital and national reporting. An important objective of this work is to support improvements in the accuracy and utility of these statistics. There is large uncertainty in the estimates resulting from country differences in reporting methodology, inconsistent case definitions and potentially biased or flawed reporting. These are, however, the best available data. The present study proposes a practical methodology to incorporate admission rates for severe malaria into estimates of malaria mortality using the WMR data, and the results should be regarded with caution given the uncertainties surrounding WMR estimates. Low reported admission rates in the WMR may reflect poor access to care or incomplete reporting, or both. Unexpectedly high admission rates, on the other hand, could be a consequence of laxer definitions of severe malaria. A direct measure of reporting biases, even if thought to be considerable, has not been be undertaken in this work due to the lack of alternative data.

Each year the WMR generally reports health facility data and estimates of incidence and mortality for all sub-Saharan African countries. Only 41 countries were included in the present study due to missing data in the WMR for some countries (Equatorial Guinea, South Sudan, Cabo Verde and Swaziland) or missing parasite prevalence distributions from the MAP (South Africa).

Alternative estimates to those in WMR, and also estimates of other parameters not available from WMR are derived using the *OpenMalaria* microsimulation models [13]. The *OpenMalaria* platform supports an ensemble of stochastic, individual-based, simulation models of malaria in humans [13, 17, 18] that can be used for calibrating different malariological indices against each other [19]. This includes sub-models of infection of humans [20], incidence of morbidity including severe and in-patient cases and mortality [14].

To capture effects of the different malaria transmission settings and health care systems for each country, these models were linked to population surfaces from WorldPop [21], national level estimates of effective coverage of treatment for uncomplicated malaria [9] (*E*_14_, based on survey data with 14-day recall periods) and high spatial resolution posterior distributions of the *P. falciparum* prevalence for 2–10 year olds (*Pf*PR_2–10_) for 2014 from the Malaria Atlas Project (MAP)  [16]. Distributions of entomological inoculation rates (EIR) were estimated using these *Pf*PR_2–10_ distributions, as described previously [22]. Simulations were performed using six of the *OpenMalaria* ensemble models (capturing heterogeneity in immunity decay, transmission and co-morbidities) for each country using these country-specific inputs of EIR, population demographics, and effective treatment for uncomplicated clinical disease, as described previously [22], and detailed in the Additional file 1. The value for the proportion of severe cases that are admitted for in-patient care was assumed to be as previously estimated ($\mu = \mu_{0} = 0.48$) [14], which also results in untreated or out-patient severe cases to have approximately twice the risk of dying as do severe malaria cases that are admitted (odds ratio: $\bar{\varphi }_{1} = 2.1$ [14]). Simulation outputs included yearly incidence of total uncomplicated ($\bar{U}_{t}$) and severe ($\bar{S}_{t}$) malaria clinical cases, as well as malaria deaths ($\bar{D}_{{t,\mu_{0} ,\overline{\varphi }_{1} }}$. All model-based deaths in this work are deaths directly attributable to malaria, and indirect deaths [14] associated with co-morbidities also estimated by *OpenMalaria* are not considered.

### Case fatality rates

The public health consequences of severe malaria depend on the proportion of cases that die. This proportion, referred to as the case fatality rate (CFR), depends on whether the patient receives in-patient care.

The proportion of malaria in-patients who die (the CFR, $\hat{Q}_{h}$) estimated from WMR data is dependent on reported incidence of in-patient deaths ($\hat{D}_{h}$) and in-patients, $\hat{C}_{h}$, namely:

1 $$\hat{{Q}}_{{h}} = \frac{{\hat{{D}}_{{h}} { }}}{{\hat{{C}}_{{h}}{ }}}$$

An independent, and age-dependent, estimate of the hospital CFR is provided by Reyburn et al. [23] for Tanzania. This function, (with age-weighted average $\overline{Q}_{h}$), is an input used in calibration of *OpenMalaria* models, as previously detailed [14]. In *OpenMalaria*, only severe malaria cases are simulated as being admitted, thus the hospital CFR ($\overline{Q}_{h}$) in *OpenMalaria* is:

2 $$\overline{{Q}}_{{h}} = \frac{{\overline{{D}}_{{h}} { }}}{{\overline{{S}}_{{h}} { }}}$$

In *OpenMalaria* parameterizations that assume the proportion of severe cases admitted is $\mu_{0} = 0.48$, then the odds ratio of dying in the community compared to in-patient was estimated to be double ($\overline{\varphi }_{1} = 2.1$ [14]) leading to a CFR for severe disease in the community of:

3 $$\overline{{Q}}_{{{c,}\overline{{\varphi }}_{{1}} }} = \frac{{\overline{{\varphi }}_{{1}} \overline{{Q}}_{{h}} }}{{{1 + }\overline{{\varphi }}_{{1}} \overline{{Q}}_{{h}} { - }\overline{{Q}}_{{h}} }}$$

### Fraction and number of in-patients with severe disease

The fraction of admissions that have severe disease is a potentially important indicator of the appropriateness of admission criteria, but cannot be directly estimated from WMR because admissions are not differentiated into severe cases and uncomplicated ones. Hence, direct estimates of country-specific incidence of severe disease among in-patients ($\hat{S}_{h}$) are not available from WMR, however, it is possible to calculate $\hat{S}_{h}$ using estimated case fatality rates.

The in-patient case fatality rate from *OpenMalaria*, $\bar{Q}_{h}$, can be applied also to the severe cases in WMR so that:

4 $$\frac{{\overline{{D}}_{{h}} }}{{\overline{{S}}_{{h}} }} = \frac{{\hat{{D}}_{{h}} }}{{\hat{{S}}_{{h}} }}= \overline{{Q}}_{{h}}$$

and hence, the implicit number of severe cases among the admissions reported in WMR, $\hat{S}_{h}$, is obtained as:

5 $$\hat{S}_{h} = \frac{{\hat{D}_{h} }}{{\overline{Q}_{h} }}$$

Let *r* be the ratio of severe to total cases in a given setting, i.e., the fraction of in-patients that are severe is:

6 $$r_{h} = \frac{{S_{h} }}{{C_{h} }} = \frac{{S_{h} }}{{S_{h} + U_{h} }}$$

In *OpenMalaria*, the admission of uncomplicated cases as in-patients is assumed to be irrelevant, thus $\overline{U}_{h} = 0 \Rightarrow \overline{r}_{h} = 1$. However, since this assumption does not hold for WMR data, that is $\hat{U}_{h}$ for some countries is non-negligible, in general $\hat{ r}_{h}$ < 1, and the best-estimate of the proportion of in-patient cases that are severe is:

7 $$\hat{r}_{h} = \frac{{\hat{S}_{h} }}{{\hat{S}_{h} + \hat{U}_{h} }}$$

Substituting for WMR reported number of severe cases admitted (Eq. 5) results in an estimated fraction of in-patients with severe disease, as the ratio of in-patient CFRs from WMR and from the *OpenMalaria* predictions, namely:

8 $$\hat{r}_{h} = \frac{{\hat{Q}_{h} }}{{\overline{Q}_{h} }}$$

### Estimates of the total incidence of severe disease and the proportion admitted

The total incidence of severe disease, $S_{t}$, is an important measure of burden but has not previously been estimated in national statistics and is not directly available from WMR because admissions are not classified by severity and the number of severe cases in the community is not available. To estimate the total incidence of severe disease and the proportion that are admitted we took two approaches that combine reported national level malaria incidence from WMR and model estimates:

1. Approach 1: *OpenMalaria* generally simulates higher mortality rates than those in WMR. The *OpenMalaria* estimate of the overall incidence of severe malaria, $\overline{S}_{t}$, might be higher than contemporary rates because it is parameterized using data from historical studies when co-infections, which contribute to the pathogenesis of severe disease, were more frequent than they are now [14].

The overall ratio, $\rho$, of the number of deaths per year in WMR ($\hat{D}_{t}$) [allowing for the national population (*N*)], to that predicted by *OpenMalaria* ($\overline{D}_{{t,\mu ,\overline{\varphi }_{1} }}$) is:

9 $$\rho_{{\mu ,\overline{\varphi }_{1} }} = \frac{{\mathop \sum \nolimits N \hat{D}_{t} }}{{\mathop \sum \nolimits N\overline{D}_{{t,\mu ,\overline{\varphi }_{1} }} }}$$

where the summations are over all 41 countries, and the value of $\mu$ for each country is determined via an iterative algorithm as described in the Additional file 1. This ratio can be used for scaling the incidence of model estimates of severe disease to WMR, in principle allowing for the decrease in comorbidity over time:

10 $$S_{PB} = \rho \overline{S}_{t}$$

This adjusted estimates of severe disease incidence provides the *prediction*-*biased* estimate, $\mu_{PB}$, of the fraction of all severe cases that are admitted, namely:

11 $$\mu_{PB} = \frac{{\hat{S}_h}}{{S_{{PB}} }} = \frac{{\hat{D}_h }}{{\overline{Q}_h\rho\overline{S}_t}} = \frac{{\hat{r}_h \hat{C}_h} }{{\rho\overline{S}_t }}$$

2. Approach 2: A second set of estimates was derived by using the reported or inferred number of WMR deaths and assuming the applicability of the community case fatality rate from *OpenMalaria*, $$\overline{Q}_{c}$$. These are the *deaths*-*adjusted* estimate of total severe malaria ($$S_{t} )$$ obtained by expanding $$S_{t}$$ as the sum of $$S_{h} )$$ and $$S_{c}$$; the former is by definition equal to $${r_{h}}{C_{h}}$$ and the latter follows from the fact that cases can be computed as the ratio of deaths and the case fatality rate:

12 $$S_{DA} = \frac{{\hat{D}_{c} }}{{\overline{Q}_{c} }} + \frac{{\hat{D}_{h} }}{{\overline{Q}_{h} }} = \frac{{\hat{D}_{c} }}{{\overline{Q}_{c} }} + \hat{r}_{h} \hat{C}_{h} ,$$

and the *deaths*-*adjusted* estimate, $\mu_{DA}$, of the fraction admitted to in-patient care is:

13 $$\mu_{DA} = \frac{{\hat{S}_h }}{{S_{DA} }} = \frac{{\hat{D}_h }}{{\left( {\overline{Q}_{h} /\overline{Q}_{c} } \right)\hat{D}_{c} + \hat{D}_h }} = \frac{{\hat{r}_h \hat{C}_h }}{{\hat{r}_h \hat{C}_h + \hat{D}_{c} /\bar{Q}_{c} }}$$

### Mortality estimates adjusting for access to in-patient care

The overall incidence of malaria deaths *D*_*t*_, depends on the proportion of severe cases receiving in-patient care, *μ*, because of the higher mortality (CFR: $Q_{c}$) of severe malaria cases who are not admitted [14], so that:

14 $$D_{t} = \mu Q_{h} S_{t} + \left( {1 - \mu } \right)Q_{c} S_{t} .$$

Both in-patient and community malaria mortality were re-estimated based on each of the estimates of the country-specific proportions of severe cases receiving in-patient care ($\mu_{PB}$ and $\mu_{DA}$).

Neither WMR estimate, $\hat{D}_{t}$, nor the *OpenMalaria* estimate, $\overline{D}_{{t,\mu_{0} }}$, of overall mortality rates, allow for variation in $\mu ,$ but improved country specific estimates of overall malaria mortality can be obtained using $\mu_{PB}$ or $\mu_{DA}$ for the proportion of severe cases receiving in-patient care in each case using the estimates of case fatality rates from *OpenMalaria*:

15 $$\hat{D}_{PB} = \rho \overline{S}_{t} \left( {\mu_{PB} \overline{Q}_{h} + \left( {1 - \mu_{PB} } \right)\overline{Q}_{c} } \right),$$

16 $$\hat{D}_{DA} = \rho \overline{S}_{t} \left( {\mu_{DA} \overline{Q}_{h} + \left( {1 - \mu_{DA} } \right)\overline{Q}_{c} } \right),$$

where $\hat{D}_{DA}$ and $\hat{D}_{PB}$ are scaled with the factor $\rho$ to the average mortality in WMR.

### Potential public health impact of improving access to in-patient care

The potential reduction in mortality, *R*, that would be achieved by increasing access to in-patient care for all severe malaria cases, is obtained by taking the difference between estimated mortality, $\hat{D}$, with current estimated levels of access to in-patient care ($\mu_{PB}$ or $\mu_{DA}$) and the mortality obtained by assuming 100% access to in-patient care ($\mu = 1$), namely:

17 $$\hat{R}_{PB} = \rho \overline{S}_{t} \left( {1 - \mu_{PB} } \right)\left( {\overline{Q}_{c} - \overline{Q}_{h} } \right),$$

18 $$\hat{R}_{DA} = \rho \overline{S}_{t} \left( {1 - \mu_{DA} } \right)\left( {\overline{Q}_{c} - \overline{Q}_{h} } \right).$$

### Sensitivity analyses

We undertook a sensitivity analysis to examine how estimates of severe disease and mortality depend on assumptions about the odds ratio of dying in the community compared to in-patient ($\varphi_{1}$) and the related parameter of proportion of severe cases receiving in-patient care ($\mu$). The analysis is described in the Additional file 1.

## Results

The burden and access statistics described in the results were computed for each of the 41 malaria endemic countries. Malaria burden estimates from the *World Malaria Report* and the *OpenMalaria* simulations are given in the Supporting Information Table 2. These are derived from the national level distributions of EIR for 2014 detailed in the Additional file 1: Figure S1 and Table S1.

**Table 2:** Country specific access to care, case fatality rates, and in-patient malaria incidence

	Access to care				Cases fatality rate (CFR)				In-patient cases (per 100,000 person per year)	
	Effective access to care for uncomplicated cases		Proportion admitted as in-patients		Community	In-patient		In-patient fatality rate ratio	Uncomplicated	Severe
					OM	WMR	OM	*WMR/OM*		
Country	Code	$E_{14}$	$\mu_{PB}$	$\mu_{DA}$ ^a^	$\overline{Q}_c$	$\hat{Q}_{h}$	$\overline{Q}_{h}$	$\hat{r}_{h}$	$\hat{U}_{h}$ ^b^	$\hat{S}_{h}$ ^b^
Angola	ago	0.49	0.74	0.57 (0.43;0.78)	0.15	0.02	0.08	0.3	689.7	296.1
Benin	ben	0.3	0.43	0.45 (0.36;0.59)	0.15	0.02	0.08	0.26	628.9	225.4
Botswana	Bwa	0.71	0.11	1 (–;–)	0.16	0.14	0.08	1.74	0	11.9
Burkina Faso	bfa	0.34	0.78	0.49 (0.29;0.63)	0.14	0.01	0.07	0.16	2209.5	427.2
Burundi	bdi	0.38	0.86	0.96 (0.68;1)	0.16	0.02	0.08	0.24	1081.3	337.5
Cameroon	cmr	0.26	0.53	0.63 (0.47;0.91)	0.15	0.01	0.08	0.12	1829	240.1
Chad	tcd	0.1	0.65	0.35 (0.26;0.68)	0.17	0.04	0.09	0.42	204.9	146.2
Central Afr Rep.	caf	0.17	0.34	0.28 (0.22;0.37)	0.15	0.02	0.08	0.26	483.6	167.9
Comoros	com	0.27	0	0 (0;0)	0.15	0	0.08	0	136.2	0
Congo	cog	0.38	0.16	0.28 (0.2;0.95)	0.15	0.01	0.08	0.13	490.1	74.9
Rép. Dém. du Congo	cod	0.26	0.86	0.67 (0.51;0.87)	0.15	0.03	0.08	0.33	888.4	435.1
Côte d’Ivoire	civ	0.25	0.2	0.22 (0.18;0.29)	0.15	0.03	0.08	0.4	184	124.1
Djibouti	dji	0.47	1	0.71 (–;–)	0.16	0.02	0.09	0.28	96.2	37.4
Eritrea	eri	0.08	0.1	0.2 (0.1;1)	0.18	0	0.09	0.04	72.1	3.2
Ethiopia	eth	0.14	0.21	0.06 (0.02;0.94)	0.18	0.01	0.1	0.07	31.5	2.3
Gabon	gab	0.4	0.26	0.59 (0.47;1)	0.15	0.01	0.08	0.07	1539	121.1
The Gambia	gmb	0.37	0.53	0.43 (0.3;1)	0.17	0.03	0.09	0.34	193.1	97.8
Ghana	gha	0.31	0.2	0.26 (0.21;0.53)	0.15	0.01	0.08	0.07	1498.4	106.7
Guinea	gin	0.21	0.21	0.18 (0.15;0.25)	0.15	0.01	0.08	0.13	800.3	115.6
Guinea Bissau	gnb	0.29	0.74	0.68 (0.52;1)	0.16	0.03	0.09	0.32	498.6	231.5
Kenya	ken	0.35	0.04	0.09 (0.07;0.31)	0.15	0.02	0.08	0.28	33.7	13.1
Liberia	lbr	0.42	1	1 (0.88;1)	0.14	0.08	0.07	1.08	0	696.9
Madagascar	mdg	0.22	0.12	0.28 (0.13;1)	0.17	0.06	0.09	0.68	12.5	26.4
Malawi	mwi	0.4	0.71	0.72 (0.57;1)	0.15	0.05	0.08	0.64	189.4	339.2
Mali	mli	0.2	0.31	0.2 (0.16;0.26)	0.15	0.04	0.08	0.48	198.3	179.7
Mauritania	mrt	0.22	0.04	0.03 (0.02;0.14)	0.17	0	0.09	0.02	326.2	5.4
Mozambique	moz	0.38	0.3	0.32 (0.26;0.5)	0.15	0.03	0.08	0.45	188.6	156.3
Namibia	nam	0.44	0.13	1 (–;–)	0.16	0.04	0.08	0.5	30.5	30.9
Niger	ner	0.39	0.38	0.36 (0.27;0.53)	0.15	0.01	0.08	0.18	831	180.6
Nigeria	nga	0.32	0.08	0.09 (0.08;0.14)	0.15	0.01	0.08	0.09	478.7	44.8
Rwanda	rwa	0.54	0.19	0.28 (0.19;1)	0.16	0.04	0.08	0.54	45.3	52.9
São Tomé e Prìncipe	stp	0.54	0	0 (–;–)	0.15	0	0.08	0	223.8	0
Senegal	sen	0.32	0.15	0.2 (0.14;0.86)	0.17	0.04	0.09	0.45	47.6	38.5
Sierra Leone	sle	0.5	1	0.53 (0.4;0.66)	0.14	0.15	0.07	2.1	0	618
Somalia	som	0.08	0.02	0.01 (0.01;0.49)	0.17	0.01	0.09	0.12	10.7	1.5
Sudan	sdn	0.25	0.2	0.39 (0.22;1)	0.17	0.01	0.09	0.07	320.4	23
Tanzania	tza	0.46	0.33	0.48 (0.37;1)	0.16	0.03	0.08	0.31	284.4	126.4
Togo	tgo	0.32	0.41	0.4 (0.33;0.55)	0.15	0.04	0.08	0.48	246.7	224.7
Uganda	uga	0.66	0.59	0.63 (0.51;1)	0.15	0.01	0.08	0.13	1438.3	207.3
Zambia	zmb	0.59	0.66	0.65 (0.51;1)	0.15	0.02	0.08	0.28	701.6	271.7
Zimbabwe	zwe	0.32	0.16	0.26 (0.13;1)	0.17	0.05	0.09	0.6	20.4	30.1

^a^Ranges represent the estimates using the lower and upper bound of deaths estimates in WMR

^b^ $\hat{U}_h$ estimated as: $\hat{U}_h = \hat{C}_h - \hat{S}_h$; $\hat{S}_h$ estimated as $\hat{S}_h = \frac{{\hat{D}_h }}{{\overline{Q}_h }}$

**Fig. 1:**
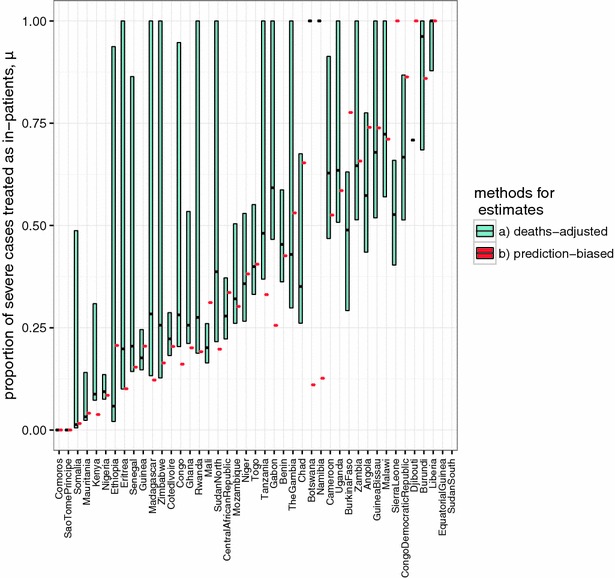
Estimates of proportion of severe cases receiving in-patient care: country estimates of the proportion of severe cases receiving in-patient care, $\mu$, by method of estimation. *Colour* indicates method with the prediction biased estimate ($\mu_{PB}$) in *orange* and the deaths-adjusted estimate ($\mu_{DA}$) in *green*. For the deaths adjusted estimate the bar indicates the min and max range, and *black* the mean

The derived country specific values for access to care and case fatality rates are given in Table 2 and Fig. 1. The in-patient CFRs from WMR, $\hat{Q}_{h}$, vary considerably, ranging from 0 (Eritrea and Mauritania) to 15% (Sierra Leone). In contrast, the *OpenMalaria* in-patient case fatality rates, $\overline{Q}_{c}$, are very similar across countries, reflecting the fact that they depend only on differences in the modelled age-distributions of severe malaria cases. The *OpenMalaria* CFRs are higher than those from WMR, with the exceptions of three countries, Liberia, Botswana and Sierra Leone, for which the ratio of severe to clinical cases is greater than 1 ($r_{h} > 1$). For these three countries we assumed for subsequent calculations that all in-patients have severe disease, ($r_{h} = 1$). The estimates of the proportions of in-patients that are severe, $r_{h}$, range from 10 to 70% for most of the countries (Table 2).

Low in-patient case fatality rates ($\hat{Q}_{h}$) are to be expected in those countries where uncomplicated cases are frequently admitted to in-patient facilities. Admission of uncomplicated cases was not considered in the simulation models so it does not affect the *OpenMalaria* CFR, $\overline{Q}_{h}$. However, in WMR, very high rates of in-patient mortality ($\hat{Q}_{h}$) can derive from poor quality of case-management, or arise because patients have difficulty accessing care until it is too late (both reflecting poor health system performance). High in-patient CFR, $\hat{Q}_{h}$, values might also arise because of strict referral or admission criteria, indicated by a high ratio of severe to uncomplicated cases among the admissions, $r_{h}$. If $r_{h}$ could be estimated independently, then a high value of $r_{h}$ associated with a low value of $\hat{Q}_{h}$ would indicate an efficient system of tertiary care for severe malaria, but unfortunately we have no estimate of $r_{h}$ that is independent of the CFRs. The ratio of severe to uncomplicated cases, $r_{h}$, is not correlated with the quality of care for uncomplicated malaria, as measured by *E*_14_ (Pearson correlation coefficient 0.39 [0.098–0.63], Additional file 1: Figure S4).

Data gaps in WMR [5] affect estimates of the proportion of severe disease cases admitted for several countries. In particular, for South Sudan and Equatorial Guinea, WMR does not contain data on in-patient mortality and thus the proportion of severe cases admitted could not be estimated for those two countries. For Botswana, Djibouti and Namibia, all countries with low malaria incidence, WMR gives estimates of total deaths as an upper bound, lower than the number of in-patient deaths reported, so for these countries the in-patient report provides the best estimate of total mortality and the proportion of severe cases that are admitted is assumed to be 100%. Similarly, in both Comoros and São Tomé and Príncipe the in-patient deaths are reported to be 0 which leads to estimates of μ_DA_ = μ_PB_ = 0.

**Fig. 2:**
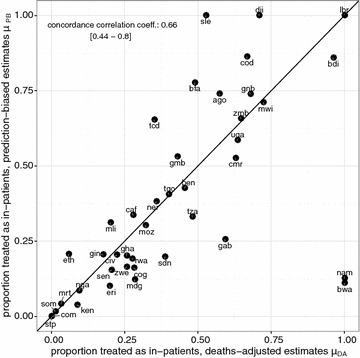
Relationship between mean estimates of the proportion of severe cases treated as in-patients for the two estimation methods. Country specific mean estimates of the prediction biased estimate of severe access to care ($\mu_{PB}$) is shown on the vertical axis and the mean deaths-adjusted estimate ($\mu_{DA}$) on the horizontal axis. The concordance correlation co-efficient was estimated as 0.66 with a confidence interval of [0.44–0.8] indicating close agreement between the two estimates. The *black line* indicates $\mu_{PB}$ = $\mu_{DA}$ line, and each country is indicated via their country code (Table 2)

In general, the *OpenMalaria* simulations predicted much higher mortality rates than those in WMR, with the overall ratio calculated to be $\rho = 0.45$. Estimates of the proportion admitted (both of $\mu_{PB}$ and $\mu_{DA}$ computed by reweighting using $\rho$), for countries with complete data cover the whole range from little more than 0–100% (Fig. 1). For many countries $\mu_{DA}$ is extremely uncertain, as illustrated by the uncertainty bounds, which correspond to the upper and lower bounds for the estimated mortality rates from WMR. Corresponding bounds are not available for $\mu_{PB}$ but this does not mean that either of these estimates is more accurate than the other. Estimates of $\mu_{DA}$ and $\mu_{PB}$ are strongly correlated with each other (Fig. 2), but by no means identical. Neither estimate of access to care for severe disease is strongly correlated with effective access to care for uncomplicated malaria ($E_{14}$; Additional file 1: Figure S2) and nor is there any clear relationship of either measure with DTP3 vaccination coverage (a frequently used measure of health system performance; Additional file 1: Figure S3). Correlation to other measures such as the transmission with the mean EIR (Pearson coeff. −0.031 [−0.033–0.28] and 0.26 [−0.046–0.53] for deaths-adjusted and prediction-biased estimates respectively), the IQR of the EIR distribution (Pearson coeff. −0.080 [−0.23–0.38] and 0.40 [−0.11–0.64] for deaths-adjusted and prediction-biased estimates respectively), or the correlation to the national GDP (Pearson coeff. 0.36 [0.025–0.61] and −0.20 [−0.50–0.14] for deaths-adjusted and prediction-biased estimates respectively) have been assessed, but no correlation has been found.

**Fig. 3:**
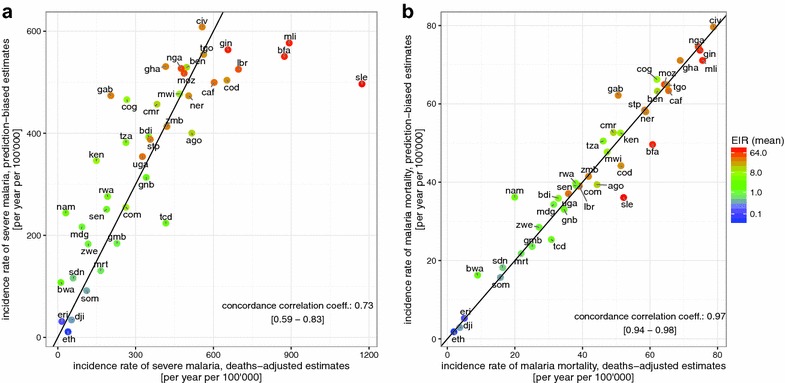
Predicted national levels of severe incidence and malaria mortality rates. **a** Severe incidence (per year per 100,000) and (**b**) malaria mortality (per year per 100,000). In both panels, the *horizontal axis* indicates predicted national levels assuming the deaths-adjusted estimate of the proportion of severe cases treated as in-patients. The* vertical axis* indicates predicted national levels when assuming the prediction-biased estimate of the proportion of severe cases treated as in-patients. Mean EIR for each country is indicated by colour, with *red high* and *blue low*. The concordance correlation co-efficient was estimated as 0.73 with a confidence interval of [0.59–0.83] in **a**, and 0.97 with a confidence interval of [0.94–0.98] in **b**, indicating close agreement between the two estimates. Each country is indicated via their country code (Table 2) and the *black line* represents the line of equality between the two estimates

Corresponding to the estimates of $\mu_{PB}$ and $\mu_{DA}$ are values of the incidence of severe disease, $S_{DA}$ and $S_{PB}$ (Fig. 3a) which vary over a large range, showing a plausible increase with malaria endemicity (Fig. 3a). Estimated $S_{DA}$ and $S_{PB}$ are strongly correlated, but not identical, with the deaths-adjusted estimates $S_{DA}$ substantially higher in some high endemicity countries, in particular Sierra Leone, Burkina Faso and Mali.

**Fig. 4:**
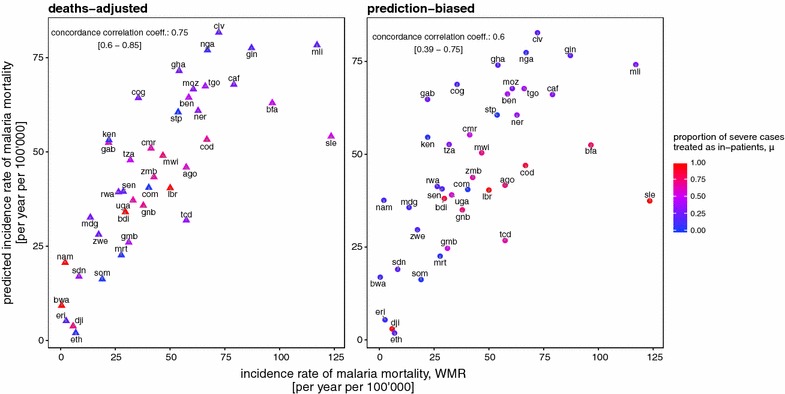
Predicted national levels of mortality rates compared with WMR estimates. In both panels, the *horizontal axis* indicates the WMR estimates of national mortality rates (per year per 100,000). The* vertical axis* indicates predicted national levels of malaria mortality when assuming; **a** the prediction-biased proportion of severe cases treated as in-patients, **b** the deaths-adjusted proportion of severe cases treated as in-patients. Mean estimates of proportion of severe cases receiving in-patient care is indicated by *colour*, with *red high* and *blue low*. The concordance correlation co-efficient was estimated as 0.74 with a confidence interval of [0.59–0.84] in **a** and 0.6 with a confidence interval of [0.38–0.75] in **b**. Each country is indicated via their country code (Table 2)

The estimates of malaria mortality rates also vary enormously between countries (Fig. 3b). The scaling of the two estimates, $\hat{D}_{PB}$ and $\hat{D}_{DA}$, ensures that on average, they are close to WMR values, and the two adjusted estimates are generally similar, both suggesting lower mortality than WMR in countries with high access to in-patient care, and higher mortality where access to in-patient care is poor (Fig. 4).

**Fig. 5:**
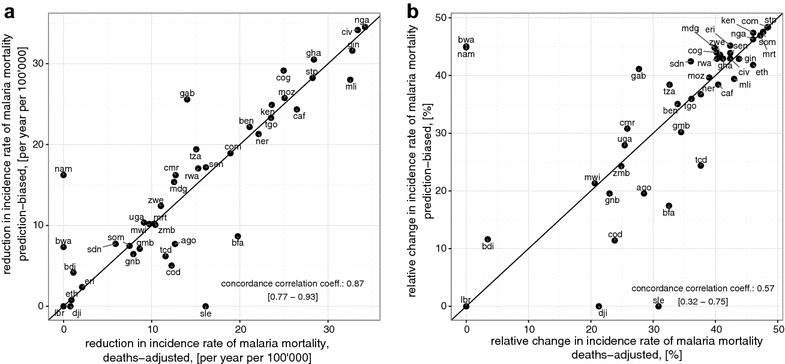
Expected national mortality reduction if access to severe in-patient treatment was universal. **a** prediction of the potential reduction in mortality rate (per year per 100,000) and (**b**) predictions of the potential reduction in mortality as a proportion of current predicted burden achieved by improving access to in-patient care. In both panels, the *horizontal axis* indicates predictions assuming the deaths-adjusted estimation method and the* vertical axis* indicates predictions assuming the prediction-biased estimation method. Each country is indicated via their country code (Table 2) and the *black line* represents the line of equality between the two estimates. In **a** the concordance correlation co-efficient was estimated as 0.87 with confidence interval of [0.77–0.93] indicating close agreement between the two mortality estimates. In **b** the concordance correlation co-efficient was estimated as 0.57 with confidence interval of [0.32–0.75] indicating moderate agreement between the two estimates

A theoretical admission rate for severe malaria of 100% would decrease malaria burden, and the deaths averted in each country if it would reach this ideal level is shown in Fig. 5a. The general pattern of the estimates of potential public health impact of improving access to in-patient care is similar to that of the estimates of the mortality rates. The two predictions of the potential number of deaths averted by improving access to in-patient care are strongly correlated (concordance correlation of 0.88 [0.78–0.93]) and both indicate that the potential gains in survival are considerable (Fig. 5a). When expressed as estimates of the proportion of malaria deaths that may be averted (Fig. 5b) a large number of countries cluster in the top right of the plot. These are countries with low estimates of access to in-patient care, for which both $\hat{R}_{PB} / \hat{D}_{PB}$ and $\hat{R}_{DA} / \hat{D}_{DA}$ approach an upper limit corresponding to $\overline{Q}_{h} / \overline{Q}_{c}$.

The sensitivity analysis (Additional file 1) of the dependence of $Q_{c}$, $\mu$, $S_{c}$ and $D_{t}$ on $\varphi_{1}$, indicated that many of these estimates are strongly related to the highly uncertain odds ratio of dying in the community compared to in-patient, $\varphi_{1}$, when the relatively well-defined parameters are fixed. This is especially the case for $Q_{c}$ which is close to linearly related to $\varphi_{1}$. If $\varphi_{1}$ is much greater than the *OpenMalaria* value of $2.1$ (and $Q_{c}$ therefore also higher than the core estimates, as in Thwing et al. [4]) then the primary estimates substantially understate the variation between countries in the incidence of severe malaria cases that are not admitted.

## Discussion

National level statistics on numbers of malaria in-patient admissions are part of routine reporting [5], but the criteria for admission of a malaria case vary considerably between countries (and probably also within countries). The analysis in this work shows that, on one hand, in most African countries far more patients are admitted than would meet strict criteria for severe (life-threatening) malaria. But, on the other hand it is likely for many countries that only a small proportion of severe cases are admitted for in-patient treatment.

Given far more patients are estimated to be admitted for in-patient care than are strictly severe malaria (defined by WHO as malaria with signs of severe illness and/or evidence of vital organ dysfunction, [24]) suggests possible inefficient use of in-patient resources and points to a need to investigate potential improvements in referral and admission practices, in many, but not all countries. This should be considered in estimating both the public health impact of in-patient care, and also the costs associated with malaria treatment in general.

Not only are severe malaria cases a small proportion of admissions, but this work also suggests that a potentially worryingly small proportion of severe cases are admitted in many African countries. There is considerable heterogeneity in access to effective care for uncomplicated malaria across the continent [9], but the national level admission rates for severe malaria estimated in this work vary even more, covering the whole range between 0 and 100%. The weak correlation at country level between access for severe and uncomplicated disease (Additional file 1: Figure S2) is perhaps surprising (because both depend on the quality of both the health-care and transport systems), but trade-offs in resource allocation between out-patient and in-patient care might play a role. Alternatively, the low reported admission rates in some countries, for instance Nigeria, could reflect incomplete reporting rather than poor access. Low reported admission rates arise when data from the private sector are omitted. As another example, in Liberia, the total deaths reported by WMR are close to the deaths reported among in-patients (2288 against 2200). This could only be correct in the unlikely scenario that almost all severe cases in the country are admitted. At both end of the spectrum there may well be substantial reporting biases.

There are a number of limitations to these international comparisons of severe malaria incidence and mortality, the key shortcoming being the dependence on data that is, at best, poorly defined, and, at worst, highly flawed. This contrasts with data on prevalence and (to a lesser extent) clinical malaria incidence, which are increasingly collected using standardized survey (DHS and MIS) methodologies. These analyses also depend on indirect inferences and simplifying assumptions made in the models. This includes not accounting for differences between countries in the average quality of care, in the efficiency of referral, and hence in timeliness of admission, as well as the assumption of uniformity in the outcome once a severe case is admitted. General understanding of the fatality rates may be substantially improved through good data on not only how many are dying or getting treated, but also whom.

The most uncertain inputs into the model relate to the proportions of severe malaria cases that are admitted, and concomitantly, to the scale of mortality due to unrecorded severe malaria in the community. The use of country specific admission rates from WMR does not help much in identifying these quantities, and further studies are needed with alternative data sources such as high resolution spatial data, length of history from in-patients, and records of treatment histories collected during verbal autopsies [25]. Such studies need to take into account the clear evidence that both disease incidence and access to in-patient care is hugely variable in space and time. *OpenMalaria* was parameterized mainly using data from the late twentieth century [14] and predicts higher mortality rates at a given level of malaria transmission than those in WMR, which are informed by more data from recent, lower general mortality settings. In this context, rescaling *OpenMalaria* severe disease incidence so that the continent-wide mortality rates matches that from WMR is coherent with overall decreases in infectious disease rates together with a contribution of co-infection to pathogenesis of severe malaria (as modelled in *OpenMalaria* [14]).

The weakness of data sources on severe disease is particularly troubling considering that averting mortality is the main reason for intervening against malaria. Since malaria is a treatable disease and treatment impacts onward transmission [22], levels of access to curative care are an important determinant of both disease burden and the public health impact of both preventive and curative interventions. If no-one is dying of malaria anyway, for whatever reason, then preventive interventions obviously cannot avert any deaths. Current mortality rates, the numbers of deaths that are already being prevented, and the numbers that could be prevented in the future by vector control, vaccination, or treatment, all depend on levels of access both to prompt and effective treatment of fevers, and to effective in-patient care for severe disease. Few analyses of either burden or the impact of preventive interventions against *P. falciparum* in Africa consider this [26, 27].

There is a clear need for effective coverage of both out-patient and in-patient care to be appropriately taken into account both in burden estimation and in analyses of the potential public health impact of improvements in both curative and preventive programmes. WMR uses levels of access to care for fevers to correct for incomplete reporting, but the national-level estimates of burden, do not allow for the effective coverage of treatment for either uncomplicated or severe disease. For high burden countries, the Child Health Epidemiology Reference Group’s verbal autopsy data-based multicause models [28, 29] (used to estimate $\hat{D}_{t}$ in this work), considers coverage of LLINs, but not of treatment. Adult mortality is inferred from child-mortality rates and endemicity, using the relationship estimated in Ross et al. [14]. For lower-burden countries, WMR multiplies the overall estimated case incidence by standard CFR estimates to obtain $\hat{D}_{t}$ but the proportion of cases treated does not adjust $\hat{D}_{t}$ downwards. In addition, not explicitly accounting for the fate of severe malaria cases that do not make it to health facilities implies that national level burden of disease statistics for *P. falciparum* malaria, such as WMR, are also highly uncertain. Recently, several geography-specific predictions of malaria intervention impact and cost-effectiveness from *OpenMalaria* have allowed for national levels of effective treatment for uncomplicated disease [30, 31], but so far not for variations in access to in-patient care. It is not clear whether even these analyses accurately capture the quantitative impact of access to effective of treatment on subsequent burden, since the strength of this relationship is not well calibrated against field data, which is also lacking.

## Conclusion

There is a pressing need for more convincing data on both admission rates for severe malaria and the total numbers of severe malaria cases in different endemic countries in Africa. The available evidence suggests that access to in-patient care for severe malaria varies considerably between countries, and the potential impacts of improvements in access to and quality of in-patient care are also strongly country-dependent. The model-based analyses proposed here offer a practicable start to incorporating severe disease rates into a common framework for comparing public health impact of preventive and curative interventions. It will allow both international and national level resource allocation decisions to make valid comparisons of the mortality impacts of different kinds of intervention packages.

## Additional file

**Additional file 1.** This file includes additional methods figures and tables of results that support and expand some of the results in the main text, but whose inclusion would detract from the main argument.

## Abbreviations

CFR: case fatality rate
DHS: Demographic and Health Surveys
EIR: entomological inoculation rate
MAP: Malaria Atlas Project
MIS: Malaria Indicator Surveys
OM: *OpenMalaria*
WMR: *World Malaria Report*
