# Supplementary material for: Incidence and admission rates for severe malaria and their impact on mortality in Africa
Source: Malar J. 2017 Jan 3;16:1. doi: 10.1186/s12936-016-1650-6 (PMC5209951; doi:10.1186/s12936-016-1650-6)
Supplement: Supplementary file 1 — Additional file 1. This file includes additional methods figures and tables of results that support and expand some of the results in the main text, but whose inclusion would detract from the main argument. [file 12936_2016_1650_MOESM1_ESM.pdf]

Supplementary material

Incidence and admission rates for severe malaria and their impact on mortality in Africa

Flavia Camponovo[1,2,§], Caitlin A. Bever[1,2,3,§], Katya Galactionova[1,2], Thomas Smith[1,2], Melissa A. Penny[1,2*]

[1] Swiss Tropical and Public Health Institute, Basel, Switzerland
[2] University of Basel, Basel, Switzerland
[3] Current address: Institute for Disease Modeling, Bellevue, WA 98005, USA
[§] These authors contributed equally to this work
* Corresponding author: Melissa A. Penny
E-mail: melissa.penny@unibas.ch

Supplementary Methods and Results

Simulation Models

Simulations were performed to obtain yearly incidence of uncomplicated ($\bar{U}_t$), severe ($\bar{S}_t$), and total ($\bar{C}_t = \bar{U}_t + \bar{S}_t$) malaria clinical cases for each country using an ensemble of six *OpenMalaria* model variants [1] (capturing heterogeneity in immunity decay, transmission and co-morbidities). In each simulation a value of $\mu = 0.48$ was used for the proportion of severe cases that are admitted, and the prediction of the number of malaria deaths ($\bar{D}_{t,0.48}$), was therefore conditional on this value (for notation see Table S1). Subsequent analyses entailed re-estimation of the value of μ.

Country-specific analysis of simulation outputs

To capture effects of country specific differences and variation in malaria transmission settings and health care systems for each country, these models were linked to population surfaces from Worldpop [2], national level estimates of effective coverage of treatment for uncomplicated malaria [3] (E_{14}, based on survey data with 14-day recall periods) and high spatial resolution posterior distributions of the *P. falciparum* prevalence for 2-10 year olds (*Pf*PR$_{2\text{-}10}$) for 2014 from the Malaria Atlas Project (MAP) [4]. For each country, the national level estimates of E_{14}, pixel-specific populations and pixel-specific posterior distributions of *Pf*PR$_{2\text{-}10}$ were used to estimate the number of people exposed at each level of prevalence and value of E_{14}, and calibration curves specific for each of the six models were used to assign corresponding EIR values to these exposed populations, as described previously [5]. This leads to the EIR distributions (non-zero EIR) for each of the 41 countries shown in Figure S1. The country-specific EIR distributions are also summarized in Table S1 listing the summary statistics arithmetic mean and geometric mean of the simulated EIR for each of the 41 countries.

Relationship between access to care for severe cases and different health care measures

In order to see how the calculated access to care for severe disease relates to other health care measure, we looked at the correlation with the effective access to care for uncomplicated cases (Figure S2) and with the DTP3 immunization coverage (a frequently used measure of health system performance; Figure S3). Neither estimate of access to care for severe disease is strongly correlated with effective access to care for uncomplicated malaria and nor is there any clear relationship of either measure with DTP3 vaccination coverage

Ratio of severe to total cases admitted as in-patients

The ratio of severe to total cases admitted as in-patients, r_h, as described in the main paper [6] and listed in Table 2 shows no correlation with the effective access to care for uncomplicated cases, E_{14}, with a concordance coefficient of 0.39 with a 95% confidence interval of [0.098–0.63] (Figure S4).

Iterative estimation of access to care

The country-specific estimates of mortality rates, D_t, in Table 2 (main paper) were obtained by applying the value of $\bar{\varphi}_1 = 2.1$ and using an iterative approach that adjusted the values of μ and the scale-factor ρ to achieve maximal consistency with WMR. These analyses fixed the number of in-patient deaths (which is determined relatively precisely from WMR) and the in-patient case fatality rate Q_h(which is was taken from Reyburn *et al* [8]). For each iteration $j = 1 \ldots . J$ (where J denotes the iteration where convergence is achieved, i.e. $\rho^{(J)} = \rho^{(J-1)}$) the following calculations were carried out:

$$\bar{D}_{t,\mu_{PB}^{(j-1)},\varphi_1} \xrightarrow{eqn.\ 9} \rho^{(j)} \xrightarrow{eqn.\ 11} \mu_{PB}^{(j)} \xrightarrow{eqn.\ 14} \bar{D}_{t,\mu_{PB}^{(j)},\varphi_1}.$$

The resulting values $\rho^{(J)}, \bar{D}_{t,\mu_{PB}^{(J)},\varphi_1}$ and $\mu_{PB}^{(j)}$ were invariant to the (scalar) starting value $\mu_{PB}^{(0)}$. The equations used to estimate $\bar{D}_t$, ρ and μ_{PB} are summarized in Table S2.

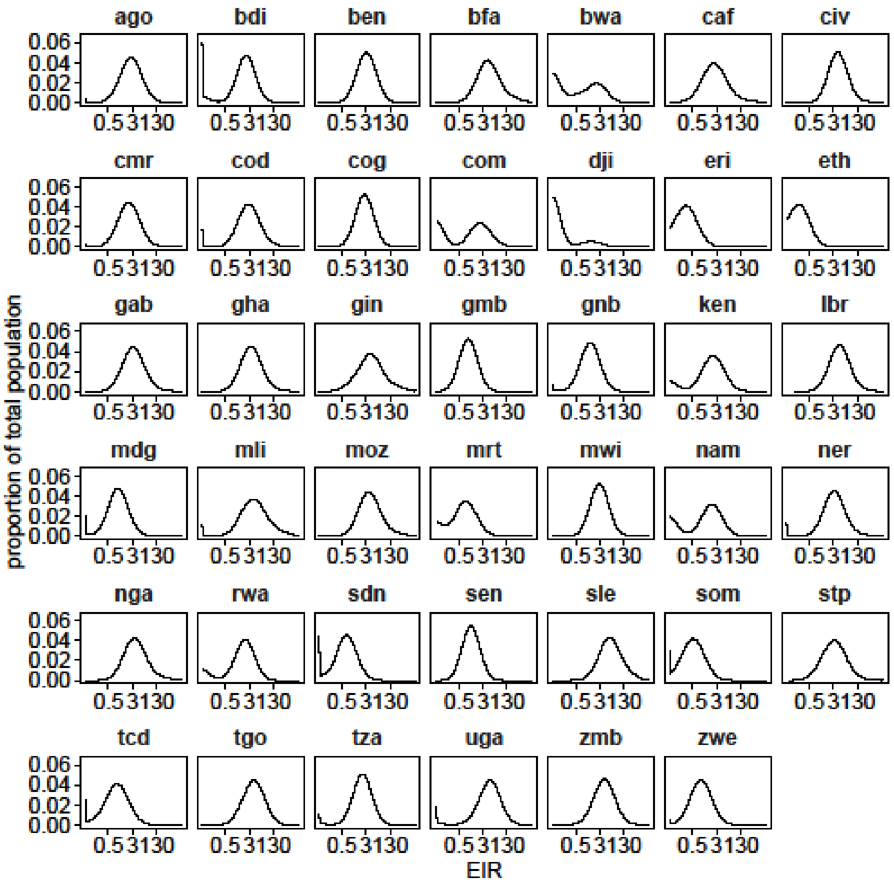

Figure S1: Histogram of the EIR distribution per country. For each of the 41 countries, a distribution of the exposed population across the EIR levels are shown (weighted by total population with unexposed population not included). Each country is indicated via their country code (for notation see main paper [1], Table 2).

Country	EIR Weighted Mean	EIR Geometric Mean	Country	EIR Weighted Mean	EIR Geometric Mean
Angola	8.75	2.3	Liberia	34.75	8.53
Benin	14.34	4.12	Madagascar	1.03	0.29
Botswana	2.32	0.04	Malawi	14.16	5.01
Burkina Faso	167.12	20.6	Mali	63.04	5.5
Burundi	5.85	1.18	Mauritania	0.67	0.08
Cameroon	7.63	1.96	Mozambique	90.63	10.29
Chad	1.43	0.28	Namibia	3.04	0.16
Central African Republic	62.83	4.6	Niger	34.37	4.57
Comoros	8.78	0.09	Nigeria	39.86	5.5
Congo	7.99	2.9	Rwanda	3.49	0.43
Congo Democratic Republic	50.45	4.13	São Tomé & Príncipe	31.52	3.62
Cote d'Ivoire	33.12	8.57	Senegal	1.6	0.62
Djibouti	0.2	0	Sierra Leone	220	35.19
Eritrea	0.11	0.02	Somalia	0.21	0.05
Ethiopia	0.05	0.01	Sudan North	0.46	0.12
Gabon	11.66	2.92	Tanzania	5.91	1.95
The Gambia	1.08	0.41	Togo	77.08	10.35
Ghana	54.89	7.54	Uganda	52.34	9.69
Guinea	102.91	8.81	Zambia	21.52	4.95
Guinea Bissau	2.38	0.75	Zimbabwe	1.05	0.27
Kenya	4.81	0.37			

Table S1: Geometric and arithmetic means of each country EIR.

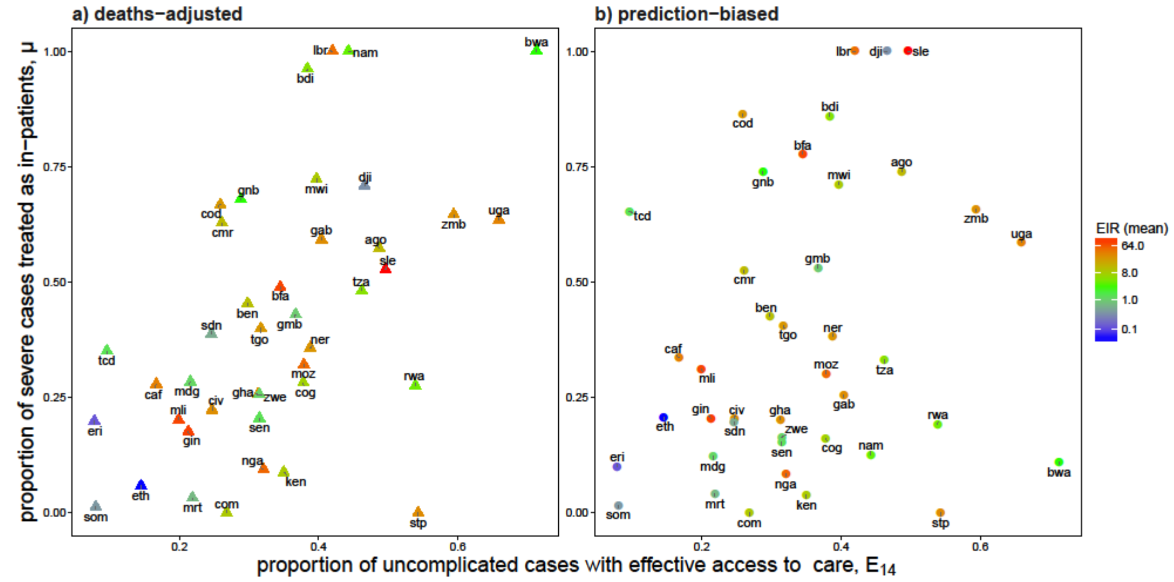

Figure S2: Relationship between mean estimates of the proportion of severe cases treated as in-patients with access to effective treatment for uncomplicated disease. The horizontal axis indicates the national level of access to effective care for uncomplicated clinical malaria in a 14 day period (E_{14}), and the vertical axis the country specific mean estimate of severe access to care. Plot a) the mean deaths-adjusted estimate (μ_{DA}), b) the prediction biased estimate of severe access to care (μ_{PB}). Mean EIR for each country is indicated by colour, with red high and blue low. The pearson correlation co-efficient was estimated as 0.52 with a confidence interval of [0.26–0.72] in a) 0.26 with a confidence interval of [-0.07–0.5] in b) . Each country is indicated via their country code (for notation see main paper [6], Table 2).

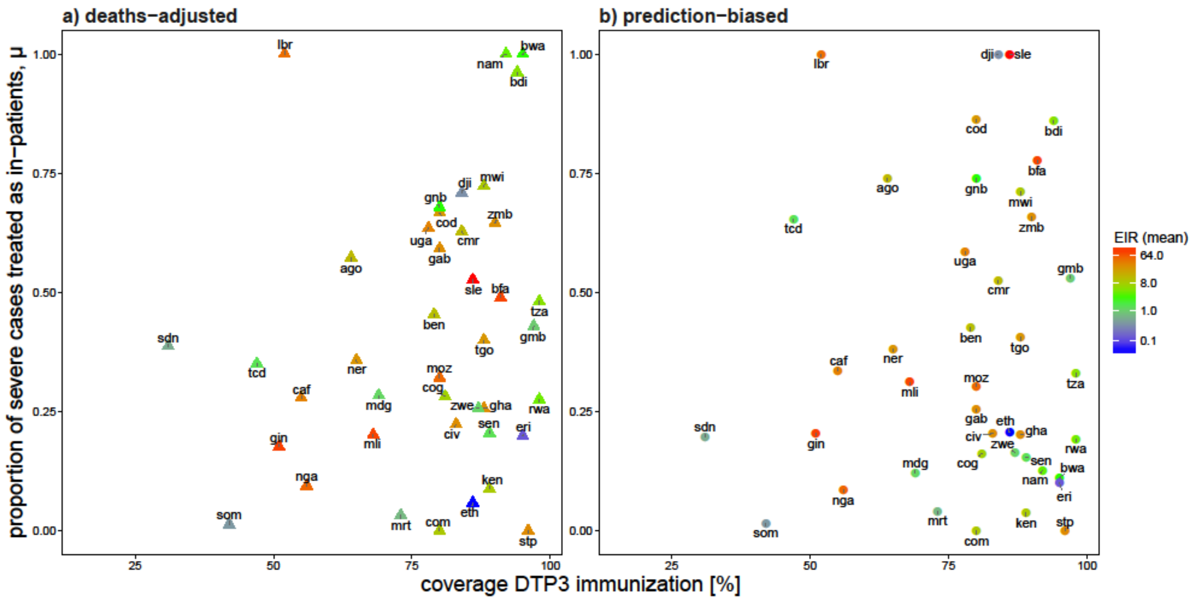

Figure S3: Relationship between mean estimates of the proportion of severe cases treated as in-patients with DTP3 vaccination coverage. The horizontal axis indicates national levels of DTP3 vaccination coverage (2015) [7] and the vertical axis the country specific mean estimate of severe access to care. Plot a) the mean deaths-adjusted estimate (μ_{DA}), b) the prediction biased estimate of severe access to care (μ_{PB}). Mean EIR for each country is indicated by colour, with red high and blue low. The pearson correlation co-efficient was estimated as 0.25 with a confidence interval of [-0.06–0.52] in a) 0.27 with a confidence interval of [-0.28–0.33] in b) Each country is indicated via their country code (for notation see main paper [6], Table 2).

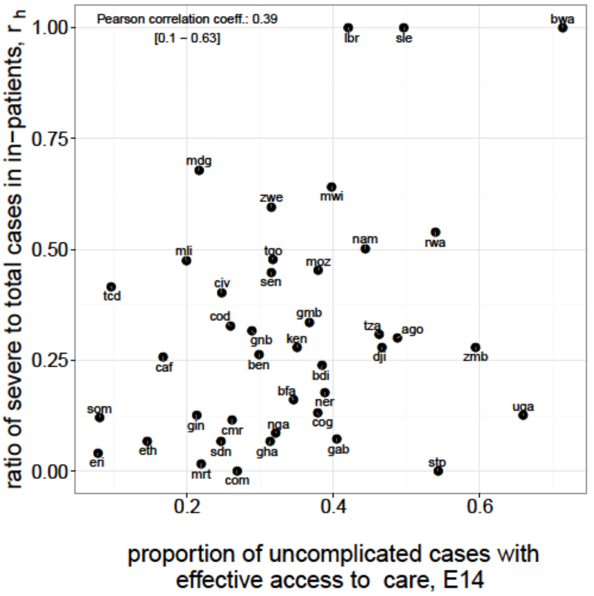

Figure S4: Relationship between the ratio of severe to total cases treated as in-patients, r_h, with effective access to care, E_{14}. The horizontal axis indicates the national level of access to effective care for uncomplicated clinical malaria in a 14 day period (E_{14}), and the vertical axis the country specific ratio of severe to total cases treated as in-patients, r_h. The concordance correlation co-efficient was estimated as 0.39 with a confidence interval of [0.098–0.63] indicating poor agreement between the two estimates. Each country is indicated via their country code (for notation see main paper [6], Table 2).

Sensitivity analyses

(i) **Implications of different community case fatality rates for country-specific estimates of the proportion of severe cases admitted (μ) and the mortality rates**

Sensitivity analyses were carried out to explore how the country specific estimates, D_t, vary when different estimates are used for φ_1. Application of the iterative algorithm (above) separately for each value of φ_1 leads to a different set of country-specific values of μ_{PB} and μ_{DA} and a new value of the scale-factor ρ used for weighting the OpenMalaria mortality rate predictions to align average mortality with WMR in the country-specific estimates, $\widehat{D}_{PB}$, and $\widehat{D}_{DA}$ (Figure S5).

The weighting factor, ρ, decreases strongly with increases in φ_1 because the OpenMalaria mortality rate estimates, $\overline{D}_{t,\mu,\varphi_1}$, become very much higher than those in WMR as the community case fatality rate, $\bar{Q}_c$ increases. The implied proportions of severe cases admitted for each country (either μ_{PB} or μ_{DA}), also increase with φ_1, since this compensates for the higher mortality of cases that are not admitted (Figure S6). With the deaths-adjusted estimate, μ_{DA}, the increase the estimated proportion admitted with φ_1 varies considerably between countries, depending on the whether the admission rate is relatively high or not. If most of the severe cases are admitted, then $\bar{Q}_c$ is less important, and μ_{DA} is less sensitive to φ_1..

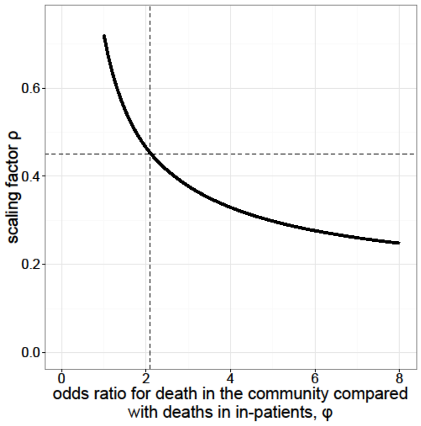

Figure S5. Value of scale-factor ρ for different values of φ_1. The y axis indicates the value of the scale-factor **ρ** taken for for each value of φ_1 (x-axis). The odds ratio for death in the community compared with death in in-patients, φ_1, varies from φ_1=1 (same odds of dying in the community as for in-patients) to $\varphi_1 = 8$. The value taking by ρ when $\varphi_1 = 2.09$ is represented by the dashed lines.

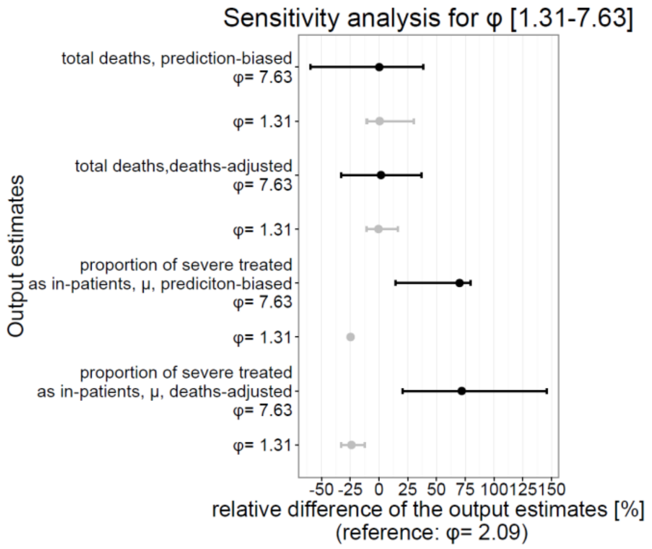

Figure S6. Sensitivity of mortality and μ estimates to value of φ_1. Estimates of the proportion of severe cases treated as in-patients, μ, and the consequent total mortality estimates, $\overline{D}_t$, are calculated for $\varphi_1$values of $\varphi_1 = 1.31$ and $\varphi_1 = 7.63$ (95% confidence interval). In the figure the average relative difference across the countries of the estimates compared to the reference when $\varphi_1 = 2.09$, are shown for both the prediction biased and deaths-adjusted method. The bars represent the minimum and maximum relative difference across the countries.

The effect of changing φ_1 on the country-specific estimates of mortality rates, $\widehat{D}_{PB}$, and $\widehat{D}_{DA}$ also varies considerably between countries (Figure S6), with those countries with the lowest admission rates showing considerably higher adjusted mortality rates with high φ_1 values, and lower adjusted mortality with low φ_1.

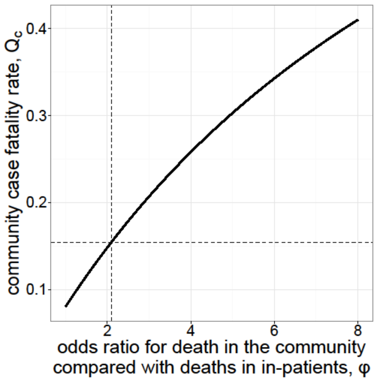

Figure S7: Dependence of Q_c on φ_1, (conditional on a typical values of $Q_h = 0.08$) . The y-axis shows the value of the community case fatality rate, Q_c, in function of φ_1 (x-axis).which is ranging from $\varphi_1 = 1$ to $\varphi_1 = 8$. The value taking by Q_c when $\varphi_1 = 2.09$ is represented by the dashed lines.

This analysis indicates that a high value of φ_1 (as suggested by Thwing *et al* [9]) would imply that our primary estimates understate the variation between countries in the incidence of severe malaria cases that are not admitted. The estimates in Table S4, of the consequent deviations in mortality rates, D_t, from WMR values, are also likely to be conservative if φ_1 in fact takes a high value.

(ii) Effect of assumed average proportions of severe cases admitted, or community case fatality rates on overall incidence of severe disease and mortality

There are no good direct estimates of how many cases of severe malaria in endemic countries fail to access appropriate care. Admission rates for severe malaria, S_h, and in-patient case fatality rates, Q_h, (such as those from Reyburn *et al* [8]) are available from research settings, as are malaria mortality rates, D_t, from health and demographic surveillance systems [10] (albeit with reservations about the validity of verbal autopsies). The mortality due to severe malaria cases that do not reach appropriate care, D_c, can be obtained by subtracting in-patient mortality rates from D_t. However D_c is the product of two quantities that cannot be estimated directly from either health facility or community survey data: the community case fatality rate, Q_c, and the incidence of such cases, S_c. For predicting program impacts, it may be essential to separate these two variables, as they differentially affect the health impact of improving access to appropriate care for severe disease. The original *OpenMalaria* parameterisation [11] used an the input value of $\mu_0 = 0.48$, [12] [13] and conditional on this, and age-dependent values of Q_h from Reyburn *et al* [8] this led to a value of $\bar{\varphi}_1 = 2.1$. In contrast, Thwing *et al* used the results of a Delphi survey to suggest that the reduction in malaria mortality in children achievable by effective management of severe disease is about 82% [5], which corresponds approximately to $\varphi_1 = 9$. It is unclear.

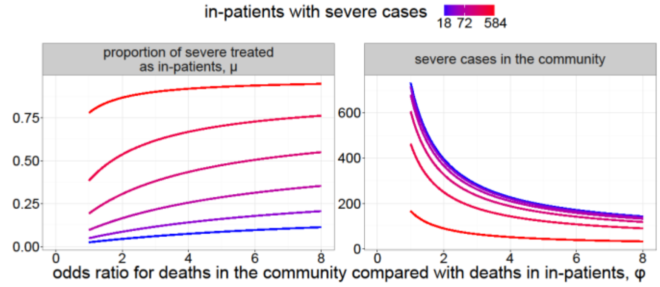

Figure S8: Dependence of μ and S_c on φ_1, conditional on typical values of S_h, D_t and Q_h, with the total number of in-patient cases with severe disease S_t varying. The y-axis shows the proportion of severe cases treated as in-patients (left-panel), μ, and the estimated incidence of severe cases in the community (right panel), S_c, in function of the value of φ_1 (x-axis). A constant value of the total deaths, $D_t = 100$, is taken to compute the outputs, and the different curves indicate the result for different incidence of in-patient severe cases, S_h, varying from low (blue) to high (red).

whether this value is compatible with plausible values of μ, Q_c and S_c.

Q_c and S_c, can be obtained conditionally on S_h, D_t and Q_h from:

$$D_t = Q_c S_c + Q_h S_h,$$

but either (i) the proportion of severe cases who are admitted, μ, is required, so that:

$S_c = \frac{(1-\mu)S_h}{\mu}$ and hence: $D_t = \frac{(1-\mu)S_h}{\mu} Q_c + Q_h S_h$, and hence:

$$Q_c = \frac{\mu(D_t - Q_h S_h)}{(1-\mu)S_h},$$

or (ii) the relative risk, or odds ratio of mortality in severe cases in the community φ_1, is required, so that, by definition: $Q_c = \frac{\varphi_1 Q_h}{1+\varphi_1 Q_h - Q_h}$ and hence the unknown quantity: $S_c = \frac{(D_t - Q_h S_h)(1+\varphi_1 Q_h - Q_h)}{\varphi_1 Q_h}$

Based on these equations, it is evident that Q_c is close to linearly related to φ_1 (Figure S7) at least for plausible values of the latter. The higher the incidence of in-patient severe disease, S_h, the higher the value of μ corresponding to any given mortality rate D_t. μ also increases with φ_1 until an upper limit is reached when $S_h = D_t/Q_h$ (Figure S8a). At this point the hospital deaths can account for all the mortality and a higher value of φ_1 is then impossible as it would imply a higher overall death rate than that assumed in the analysis. The corresponding numbers of severe cases in the community must decrease with increasing φ_1 for the same total mortality (Figure S8b). S_c is rather weakly dependent on S_h unless the latter is very high

Table S2: Variables and parameter descriptions. All the variables together with their description and corresponding equations that are used in the main paper [1] are summarized. Subscripts and accents are also specificed.

**name**	**description**	
**variables**		**Equation**
U	Incidence rate of uncomplicated clinical malaria [per 100’000 person per year]	
S	Incidence rate of severe clinical malaria [per 100’000 person per year]	Eqn 10: $S_{PB} = \rho\bar{S}_t$<br>Eqn 12: $S_{DA} = \frac{\widehat{D}_c}{\bar{Q}_c} + \frac{\widehat{D}_h}{\bar{Q}_h} = \frac{\widehat{D}_c}{\bar{Q}_c} + \hat{r}_h\hat{C}_h$
C	Incidence rate of total clinical malaria (C = U + S) [per 100’000 person per year]	
D	Incidence rate of malaria mortality* [per 100’000 person per year]	Eqn 14: $D_t = \mu Q_h S_t + (1-\mu)Q_c S_t$<br>Eqn 15: $\widehat{D}_{PB} = \rho\bar{S}_t(\mu_{PB}\bar{Q}_h + (1-\mu_{PB})\bar{Q}_c)$<br>Eqn 16: $\widehat{D}_{DA} = \rho\bar{S}_t(\mu_{DA}\bar{Q}_h + (1-\mu_{DA})\bar{Q}_c)$
μ	Proportion of severe cases treated as in-patients [-]	Eqn 11: $\mu_{PB} = \frac{\hat{S}_h}{S_{PB}} = \frac{\widehat{D}_h}{\bar{Q}_h\rho\bar{S}_t} = \frac{\hat{r}_h\hat{C}_h}{\rho\bar{S}_t}$<br>Eqn 13: $\mu_{DA} = \frac{\hat{S}_h}{S_{DA}} = \frac{\widehat{D}_h}{(\bar{Q}_h/\bar{Q}_c)\widehat{D}_c+\widehat{D}_h} = \frac{\hat{r}_h\hat{C}_h}{\hat{r}_h\hat{C}_h+\widehat{D}_c/\bar{Q}_c}$
r	Ratio of severe to total clinical cases for in-patients [-]	Eqn 7 & 8: $\hat{r}_h = \frac{\hat{S}_h}{\hat{S}_h+\widehat{U}_h} = \frac{\hat{Q}_h}{\bar{Q}_h}$.
Q	Case fatality rate [-]	Eqn 2: $\bar{Q}_h = \frac{\bar{D}_{h,\mu_0}}{\bar{S}_{h,\mu_0}}$<br>Eqn 3: $\bar{Q}_{c,\mu_0,\bar{\varphi}_1} = \frac{\bar{\varphi}_1\bar{Q}_{h,\mu_0}}{1+\bar{\varphi}_1\bar{Q}_{h,\mu_0}-\bar{Q}_{h,\mu_0}}$
R	Estimated public health impact (as malaria mortality) averted with maximal improvement to admittance of severe disease patient (μ =1) [per 100’000 person per year]	Eqn 17: $\widehat{R}_{PB} = \rho\bar{S}_t(1-\mu_{PB})(\bar{Q}_h - \bar{Q}_c)$<br>Eqn 18: $\widehat{R}_{DA} = \rho\bar{S}_t(1-\mu_{DA})(\bar{Q}_h - \bar{Q}_c)$
ρ	The overall ratio of the number of deaths per year in WMR ($\widehat{D}_t$) (allowing for the national population (N)), to that predicted by *OpenMalaria* ($\bar{D}_{t,\mu,\bar{\varphi}_1}$)	Eqn 9: $\rho_{\mu,\bar{\varphi}_1} = \frac{\sum N\,\widehat{D}_t}{\sum N\bar{D}_{t,\mu,\bar{\varphi}_1}}$

**subscripts**		**accents**	
h	indicates in-patient event	$\bar{\ }$	indicates estimation from *OpenMalaria* simulations
c	indicates event in community	$\widehat{\ }$	indicates estimation from WMR
t	indicates total events		
PB	indicates prediction-biased estimate		
DA	indicates deaths-adjusted estimate		
μ_0	indicates estimate used in OpenMalaria analysis for the proportion of severe cases treated as in-patients (usually μ_0= 0.48)		
$\bar{\varphi}_x$	Indicates estimate calculated with the odds ratio of value $\bar{\varphi}_x$		

Table S3: Malaria Burden estimates from the World Malaria Report and the *OpenMalaria* simulations

Country	Code	Total malaria incidence and mortality rate [per year per 100‘000]					In-patients malaria incidence and mortality rate [per year per 100‘000]				
		$\hat{D}_t$	$\bar{D}_{t,PB}$	$\bar{D}_{t,DA}$	$S_{t,PB}$	$S_{t,DA}$	$\hat{C}_h$	$\hat{U}_h^*$	$\hat{S}_h^*$	$\bar{S}_{h,PB}$	$\bar{S}_{h,DA}$
**Angola**	ago	57.4	38.9	43.7	395	516.7	985.9	689.7	296.1	292.3	226.4
**Benin**	ben	58.5	62.4	61.4	522.1	496.6	854.3	628.9	225.4	222.4	237
**Botswana**	bwa	0.5	16.1	8.8	106.2	11.9	6.8	0	11.9	11.7	106.2
**BurkinaFaso**	bfa	96.7	49	60	543	873.5	2636.7	2209.5	427.2	421.6	265.6
**Burundi**	bdi	29.6	35.4	32.4	387.5	350.8	1418.8	1081.3	337.5	333.1	372.7
**Cameroon**	cmr	41.3	52	48.5	451	382.3	2069.2	1829	240.1	237	283.3
**Chad**	tcd	57.4	25	30.4	220.8	416.7	351.1	204.9	146.2	144.3	77.5
**Central Afr. Rep.**	caf	79.1	62.5	64.6	493.1	602.8	651.6	483.6	167.9	165.7	137.4
**Comoros**	com	40.3	38.6	38.6	251.7	262.6	136.2	136.2	0	0	0
**Congo**	cog	35.5	65.4	61.2	459.6	266.2	565	490.1	74.9	74	129.4
**Rép. Dém. Du Congo**	cod	66.8	43.6	50.7	497.4	652.1	1323.5	888.4	435.1	429.4	331.9
**Cote d’Ivoire**	civ	72.2	78.6	77.8	600	556.7	308.1	184	124.1	122.5	133.8
**Djibouti**	dji	5.7	2.9	3.6	33.7	52.8	133.6	96.2	37.4	33.7	23.9
**Eritrea**	eri	2.5	5.2	4.9	30.9	15.9	75.3	72.1	3.2	3.1	6.1
**Ethiopia**	eth	6.9	1.8	1.9	10.9	39.2	33.8	31.5	2.3	2.3	0.6
**Gabon**	gab	21.9	61.4	49.9	467.4	204.6	1660.1	1539	121.1	119.5	276.8
**The Gambia**	gmb	31.1	23.3	24.8	181.9	227.9	290.9	193.1	97.8	96.6	78.1
**Ghana**	gha	54.1	70.1	68.1	523.6	416	1605.1	1498.4	106.7	105.3	134.3
**Guinea**	gin	87.2	72.7	73.9	556.2	655.9	915.9	800.3	115.6	114.1	98
**Guinea Bissau**	gnb	37.8	32.6	34.1	309.3	341.1	730.1	498.6	231.5	228.5	209.9
**Kenya**	ken	22.1	51.9	50.6	341.6	149	46.8	33.7	13.1	12.9	30
**Liberia**	lbr	50	38.5	38.5	518.4	696.9	643.7	0	696.9	518.4	518.4
**Madagascar**	mdg	13.6	33.9	31.1	213.6	93.1	38.9	12.5	26.4	26.1	60.6
**Malawi**	mwi	46.7	47.1	46.7	470.8	469.2	528.6	189.4	339.2	334.7	340.3
**Mali**	mli	117.1	70.2	74.6	569.1	892.4	378	198.3	179.7	177.3	114.6
**Mauritania**	mrt	27.7	21.5	21.6	129	165.7	331.5	326.2	5.4	5.3	4.2
**Mozambique**	moz	60.6	64.1	63.4	510.5	487.3	345	188.6	156.3	154.3	163.8
**Namibia**	nam	2.1	35.7	19.7	240.8	30.9	61.3	30.5	30.9	30.4	240.8
**Niger**	ner	62.8	57.2	58	467.2	504.9	1011.6	831	180.6	178.2	167.1
**Nigeria**	nga	67.1	73.6	73.3	519.9	475.5	523.5	478.7	44.8	44.2	48.9
**Rwanda**	rwa	26.5	39.2	37.5	272.6	192.1	98.2	45.3	52.9	52.2	75
**São Tomé & Prìncipe**	stp	53.7	57.7	57.7	383	356.5	223.8	223.8	0	0	0
**Senegal**	sen	28.6	38.6	37.6	247.4	188.2	86.1	47.6	38.5	38	50.7
**Sierra Leone**	sle	123.5	35.6	51.5	490.1	1173.1	293.8	0	618	490.1	258.2
**Somalia**	som	19	15.5	15.5	90.6	111.1	12.2	10.7	1.5	1.5	1.2
**Sudan North**	sdn	8.4	18	16.2	114.6	59.3	343.4	320.4	23	22.7	44.4
**Tanzania**	tza	31.8	49.9	45.6	376.9	262.8	410.7	284.4	126.4	124.7	181.2
**Togo**	tgo	66.1	64	64.2	547.2	562.2	471.5	246.7	224.7	221.8	218.7
**Uganda**	uga	33.1	36.6	35.4	349.5	326.6	1645.5	1438.3	207.3	204.6	221.9
**Zambia**	zmb	42.6	40.9	41.3	407.8	420.5	973.3	701.6	271.7	268.1	263.5
**Zimbabwe**	zwe	17.4	28.1	26.8	180.7	117.2	50.4	20.4	30.1	29.7	46.3

***: $\hat{U}_h$ estimated as: : $\hat{U}_h = \hat{C}_h - \hat{S}_h$; $\hat{S}_h$ estimated as: $\hat{S}_h = \frac{\hat{D}_h}{\bar{Q}_h}$;**

References

[1] T. Smith, G. F. Killeen, N. Maire, A. Ross, L. Molineaux, F. Tediosi, G. U. Y. Hutton, J. Utzinger, K. Dietz, and M. Tanner, “Mathematical Modeling of the Impact of Malaria Vaccines on the Clinical Epidemiology and Natural History of Plasmodium Falciparum Malaria : Overview,” *Am. J. Trop. Med. Hyg.*, vol. 75, no. Suppl 2, pp. 1–10, 2006.

[2] “WorldPop: High resolution age-structured population distribution maps,” 2015. [Online]. Available: http://www.worldpop.org.uk/.

[3] K. Galactionova, F. Tediosi, D. De Savigny, T. Smith, and M. Tanner, “Effective coverage and systems effectiveness for malaria case management in Sub-Saharan African countries,” *PLoS One*, vol. 10, no. 5, pp. 1–21, 2015.

[4] S. Bhatt, D. J. Weiss, E. Cameron, D. Bisanzio, B. Mappin, U. Dalrymple, K. E. Battle, C. L. Moyes, A. Henry, M. A. Penny, T. A. Smith, A. Bennett, J. Yukich, T. P. Eisele, P. A. Eckhoff, E. A. Wenger, O. Brie, J. T. Griffin, C. A. Fergus, M. Lynch, F. Lindgren, J. M. Cohen, C. L. J. Murray, D. L. Smith, S. I. Hay, R. E. Cibulskis, and P. W. Gething, “The effect of malaria control on *Plasmodium falciparum* in Africa between 2000 and 2015,” *Nature*, pp. 1–9, 2015.

[5] M. a Penny, N. Maire, C. a Bever, P. Pemberton-Ross, O. J. T. Briët, D. L. Smith, P. W. Gething, and T. a Smith, “Distribution of malaria exposure in endemic countries in Africa considering country levels of effective treatment.,” *Malar. J.*, vol. 14, no. 1, p. 384, 2015.

[6] F. Camponovo, C. A. Bever, N. Maire, K. Galactionova, T. A. Smith, and M. A. Penny, “ Incidence and admission rates for severe malaria and their impact on mortality in Africa.”

[7] World Health Organization, “WHO vaccine-preventable diseases: monitoring system 2016 global summary,” 2015. [Online]. Available: http://apps.who.int/immunization_monitoring/globalsummary/timeseries/tswucoveragedtp3.html. [Accessed: 01-Aug-2016].

[8] H. Reyburn, R. Mbatia, C. Drakeley, J. Bruce, I. Carneiro, R. Olomi, J. Cox, W. M. M. M. Nkya, M. Lemnge, B. M. Greenwood, and E. M. Riley, “Association of transmission intensity and age with clinical manifestations and case fatality of severe Plasmodium falciparum malaria.,” *Jama*, vol. 293, no. 12, pp. 1461–70, 2005.

[9] J. Thwing, T. P. Eisele, and R. W. Steketee, “Protective efficacy of malaria case management and intermittent preventive treatment for preventing malaria mortality in children: a systematic review for the Lives Saved Tool.,” *BMC Public Health*, vol. 11 Suppl 3, no. Suppl 3, p. S14, 2011.

[10] S. Abdullah, K. Adazu, H. Masanja, D. Diallo, A. Hodgson, E. Ilboudo-Sanogo, A. Nhacolo, S. Owusu-Agyei, R. Thompson, T. Smith, and F. N. Binka, “Patterns of age-specific mortality in children in endemic areas of sub-Saharan Africa,” *Am. J. Trop. Med. Hyg.*, vol. 77, no. SUPPL. 6, pp. 99–105, 2007.

[11] A. Ross, N. Maire, L. Molineaux, and T. Smith, “An epidemiologic model of severe morbidity and mortality caused by Plasmodium falciparum.,” *Am. J. Trop. Med. Hyg.*, vol. 75, no. 2 Suppl, pp. 63–73, 2006.

[12] S. C. McCombie, “Treatment seeking for malaria: A review of recent research,” *Soc. Sci. Med.*, vol. 43, no. 6, pp. 933–945, 1996.

[13] C. a Goodman, P. G. Coleman, and A. J. Mills, “Economic Analysis of Malaria Control in Sub-Saharan Africa,” Global Forum for Health Research, 2000.
